# Abundance and Diversity of Endolithic Fungal Assemblages in Granite and Sandstone from Victoria Land, Antarctica

**DOI:** 10.3390/life15071028

**Published:** 2025-06-27

**Authors:** Gerardo A. Stoppiello, Carmen Del Franco, Lucia Muggia, Caterina Ripa, Laura Selbmann

**Affiliations:** 1 Department of Ecological and Biological Sciences, University of Tuscia, 01100 Viterbo, Italy; carmen.delfranco@unitus.it (C.D.F.); cripa@unitus.it (C.R.); selbmann@unitus.it (L.S.); 2 Department of Life Sciences, University of Trieste, 34127 Trieste, Italy; lucia_muggia@hotmail.com; 3 Mycological Section, Italian Antarctic National Museum (MNA), 16121 Genoa, Italy; 4 Department of Earth Systems Science and Environmental Technologies (CNR-ISP), CNR-Institute of Polar Sciences, 98122 Messina, Italy

**Keywords:** Antarctica, endolithic community, granite, lichens, sandstone

## Abstract

The Antarctic continent hosts highly specialized microbial ecosystems, particularly within endolithic habitats, where microorganisms colonize the interior of rocks in order to withstand conditions that otherwise cannot support life. Previous studies have characterized the composition and abundance of these communities, as well as their different degrees of stress power; furthermore, the effect of different lithic substrates in shaping their associated bacterial assemblages has been extensively investigated. By contrast, how rock typology exerts fungal endolithic colonization still remains unexplored. In this study, we have considered and compared fungal communities inhabiting granite and sandstone rocks collected across Victoria Land, Antarctica, using high-throughput sequencing of the Internal Transcribed Spacer (ITS) region. Our analyses revealed that both rock types were dominated by Ascomycota, with a marked prevalence of lichen-forming fungi, particularly within the class Lecanoromycetes. However, granite-supported communities exhibited significantly higher species richness, likely driven by the structural heterogeneity of the substrate and the presence of fissures enabling chasmoendolithic colonization. In contrast, sandstone communities were more specialized and dominated by strict cryptoendolithic taxa capable of surviving within the rock’s pore spaces. Differential abundance analysis identified key species associated with each substrate, including the lichen *Buellia frigida* in granite and the black fungus *Friedmanniomyces endolithicus* in sandstone, two endemic species in Antarctica. Moreover, the use of presence/absence- versus abundance-based diversity metrics revealed contrasting ecological patterns; substrate type had a stronger influence on species presence, whereas geographic location more significantly shaped abundance profiles, highlighting the complex interplay between both factors in determining fungal community composition. Additionally, alpha diversity analyses showed significantly higher species richness in granite compared to sandstone, suggesting that structural heterogeneity and chasmoendolithism may promote a more diverse fungal assemblage.

## 1. Introduction

Antarctica’s ice-free areas, though scarce, represent some of the most extreme environments for life on Earth [1,2]. Among these, Victoria Land harbors extensive rock outcrops that provide the primary habitat for mainly microbial life, as the severe climatic conditions impair the settlement of vascular plants and limit biological colonization to highly specialized microorganisms [3,4]. In these environments, endolithism—microbial colonization within rock substrates—represents a key survival strategy, enabling specialized microbes to exploit a microhabitat offering more buffered conditions in airspaces or crevices of rocks, protecting them against desiccation, extreme temperatures, and direct high UV and solar exposure [5,6]. From this perspective, Antarctic cryptoendolithic communities are also considered to be the best model for studying the possibility of extant or extinct life on rocky dry and cold planets such as Mars [7,8,9]. In these ice-free areas of continental Antarctica, microbial endolithic communities, composed mainly of bacteria, lichenized and free-living fungi and microalgae, represent a prominent portion of the standing biomass, occupying approximately 4% of sandstone boulders, up to 30% of granite boulders, and 100% of sandstone cliffs [10]. They play essential roles in nutrient cycling and rock weathering, contributing to soil formation and the overall functioning of Antarctic terrestrial ecosystems [11].

Previous studies have shown that rock type significantly influences microbial community composition, providing distinct ecological niches. Fungal and bacterial diversity in Antarctic lithic substrates has been explored in various studies, e.g., [4,12], highlighting that Antarctic sandstone typically hosts cryptoendolithic communities dominated by lichenized and free-living fungi [13,14], while granite, due to its lower porosity, favors chasmoendolithic microbial assemblages, with a higher abundance of cyanobacteria that inhabit cracks and fissures [4]. A study focused on endolithic communities in granite and sandstone from Arctic regions highlighted that both bacterial and fungal community compositions were significantly correlated with the geochemical characteristics of rocks, and bacterial communities were considerably correlated with rock elements such as Mg and Ca and fungal assemblages with Fe [15]. However, the study was based on 28S rRNA genes that may only assign OTUs of very high taxonomic rank, limiting the reliability of the conclusions. The role of rock substratum in shaping bacterial assemblage composition has been recently extensively examined over a huge sampling from ice-free areas of Victoria Land, Antarctica, based on metagenomic data; the study revealed that granite supported richer and more heterogeneous communities than sandstone, and the turnover associated with elevation was also modulated by geology [16]. In the end, the role of rock substratum on fungal assemblages still remains under-examined compared to the bacterial compartment. Recent studies devoted to comparing the microbial assemblage composition of epilithic and endolithic colonization in Antarctic granite have challenged the traditional view of granite as a less hospitable substrate, revealing, for the first time, a complex lichen-dominated community within Antarctic granite [17]. This suggests that this rock typology may support a more diverse and structured fungal assemblage than previously thought, highlighting the need of a more focused comparative study to highlight the role of the two main rock typologies, granite and sandstone, for endolithic colonization in Victoria Land, Antarctica, in shaping associated fungal assemblages. To address this knowledge gap, we present the first metabarcoding analysis specifically comparing fungal diversity and composition between granite and sandstone collected across Victoria Land, Antarctica. Leveraging high-throughput sequencing, we aim to characterize fungal diversity and assess the extent to which geologically different substrates influence community richness and composition. Given the deterministic role of rock type in shaping endolithic assemblages [4,12], we hypothesize that fungal communities will differ between sandstone and granite, reflecting adaptations to the distinct microhabitats.

This study clearly indicates that granite hosts a higher myco-diversity compared to sandstone, with the presence of segregative species related to different rock substrates suggesting that, along with the strong environmental pressure and spatial/genetic isolation, geology also contributes to local diversification, leading to a peculiar biodiversity even over a relatively short geographic distance. These findings expand our understanding of fungal ecology in Antarctic rock environments by complementing previous research on bacterial diversity, shedding light on microbial life in one of the most extreme ecosystems on Earth and offering new perspectives on fungal adaptations and ecological roles in these compact and less porous substrates.

## 2. Materials and Methods

### 2.1. Dataset Acquisition, Processing and Location

The fungi amplicon-sequencing datasets of endolithic communities were retrieved from the public domain. The open access microbial sequencing raw data were downloaded from the NCBI SRA database. Using the Sratoolkit dataset, 48 endolithically colonized samples—24 granite [17] and 24 sandstone [18]—were initially taken into consideration for this study, deriving from 16 different sites located in Northern and Southern Victoria Land (NVL and SVL, respectively), Antarctica. Following library size inspection, three samples (two from Keinath and one from Kay Island) were excluded due to insufficient read counts, rendering them incomparable to the remaining dataset. Consequently, the final dataset comprised 45 samples (21 granite and 24 sandstone).

**Figure 1:**
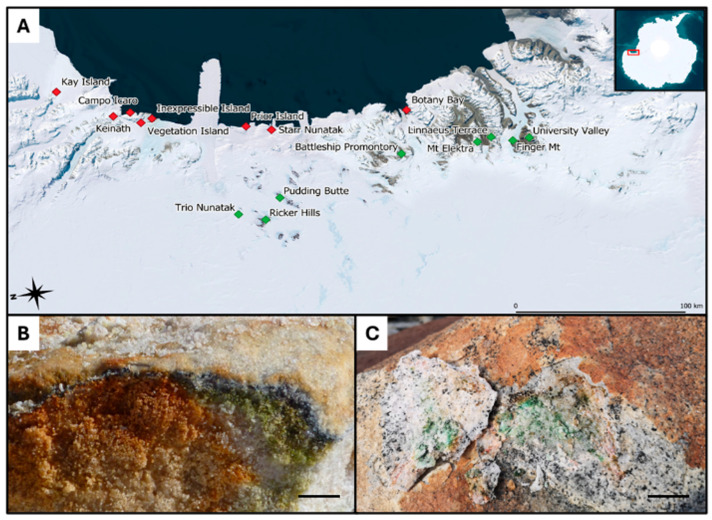
(**A**) Map displaying the 16 sites in Victoria Land (Antarctica) from which the 21 endolithically colonized granite (red squares) and the 24 endolithically colonized sandstone (green squares) samples were taken; (**B**) sandstone-associated endolithic communities (picture by Laura Selbmann); (**C**) granite-associated endolithic microbial communities with visible colonization of *Lecidea cancriformis* on the surface. Reproduced with permission from Stoppiello et al., 2025, Polar Biology; published by Springer, 2025 [17]. Scale bars: (**A**) 100 km; (**B**,**C**) scale bar 1 cm.

While granite was sampled at comparable environmental conditions in areas located along the coasts of Victoria Land and showed similar geology, sandstone was sampled in areas displaying distinct environmental conditions: some came directly from the McMurdo Dry Valleys (Southern Victoria Land, SVL), where typical sedimentary rocks of the Beacon Supergroup dominate, a formation dating back to the Devonian–Triassic (400 to 250 MYA) and composed mostly of orthoquartzite. Sandstone collected in Northern Victoria Land (NVL: Trio Nunatak, Ricker Hills and Pudding Butte, close each other), however, has a more recent origin, dating from the Triassic to the Jurassic (252 to 145 MYA) [19,20] (Figure 1). Details are provided in Table 1.

### 2.2. Bioinformatic Analysis

Samples were processed after screening for extraction contamination using Decontam [21]. Raw reads were analyzed with the Amplicon ToolKit (AMPtk) for Next-Generation Sequencing (NGS) v1.2.1 [22], alongside USEARCH [23] and VSEARCH [24]. Reads were trimmed, yielding sequences of 250 bp in length, while those shorter than 100 bp were discarded. Chimera removal was conducted using USEARCH v. 9.2.64 with default parameters. Sequence quality filtering was applied with an expected error threshold of <1.0 [22]. The dataset was clustered using DADA2 v1.6.0 with a 99% identity threshold to generate Amplicon Sequence Variants (ASVs). Additional filtering was performed, removing rare ASVs (i.e., those with fewer than five reads) and singletons, which were excluded from further analysis. Taxonomic classification was conducted using the UNITE [25] databases via the hybrid SYNTAX algorithm [23,26]. Sequences were aligned, and taxonomy was assigned to the corresponding ASVs for the ITS.

### 2.3. Statistical Analysis

To test the effectiveness of the sampling effort of the overall rock-associated fungal diversity in the studied area, species accumulation curves were calculated using the rarecurve function in the ‘vegan’ package. To investigate fungal diversity, analyses were conducted by clustering ASVs based on their substrate typology (i.e., granite vs. sandstone). Alpha diversity was assessed using the Observed, Chao1 and Shannon indices. Beta diversity was evaluated through the Jaccard index, Bray–Curtis dissimilarity index and unweighted and weighted UniFrac; PCoA analyses were carried out and tested via PERMANOVA, applying the false discovery rate (FDR) correction method. All analyses were performed using the R packages microeco 1.4.0 [27] and phyloseq 1.42.0 [28]. Kruskal–Wallis and Wilcoxon tests [29,30] were employed to determine significant differences in diversity among groups. Linear discriminant Effect Size (LEfSe) analysis was performed to identify those taxa that explain the differences between granite and sandstone mycobiota [31]. For statistical purposes, we considered only Linear Discriminant Analysis (LDA) values greater than 2. LDA was used to assess the impact of each feature’s relevance in distinguishing groups, generating an LDA score.

## 3. Results

### 3.1. Fungal Taxonomy in Colonized Sandstone and Granite

A total of 2,059,425 reads were obtained for fungi, which were clustered at 99% identity and collapsed into 1005 after removing chimeras, singletons, contaminants, and archaeal sequences and performing rarefaction at the minimum sample size of 45,765 reads. We generated species accumulation curves for the entire dataset, considering site-level data. The curve gradually plateaued toward the end of the sampling, suggesting that the number of samples analyzed was sufficient to capture the biodiversity of the studied communities (Supplementary Figure S1).

**Figure 2:**
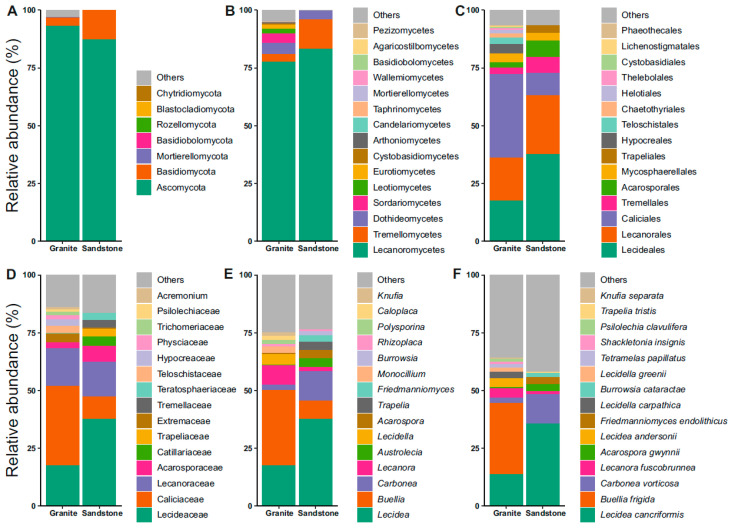
Relative abundance plots of fungi. (**A**) Phylum, (**B**) class, (**C**) order, (**D**) family, (**E**) genus, and (**F**) species reported for each of the granite and sandstone substrates.

The community analyses of the fungi revealed Ascomycota and Basidiomycota as the dominant phyla (Figure 2A). In both the granite and sandstone substrates, most of the ASVs belong to the phylum Ascomycota. Lichenized guilds were dominated by the class Lecanoromycetes in both rock types, accounting for 83% of the granite and 77.5% of the sandstone examined (Figure 2B). The other more represented classes were Dothideomycetes in granite (5%) and Tremellomycetes in sandstone (12.7%) (Figure 2B). Focusing on the orders, the most represented ones are Caliciales (36%) and Lecanorales (18.5%) for the granitic substrate, and Lecideales (37.5%) and Lecanorales (24.5%) for sandstone (Figure 2C). Moving onto the family rank, Caliciaceae (34%), Lecideaceae (17%) and Lecanoraceae (17%) were found in the granite substrate, while Lecideaceae (37%), Lecanoraceae (15%) and Caliciaceae (10%) were found in sandstone (Figure 2D). The next taxonomic rank discussed was genus, which was *Lecidea* at 17.5% and 37.5% for granite and sandstone, and *Buellia* (33%) and *Lecanora* (8.3%) in granite and *Carbonea* (13%) and *Buellia* (8%) in sandstone (Figure 2E). The last taxonomic feature analyzed was species, represented by *Buellia frigida* (31%) and *Lecidea cancriformis* (14%) in granite, and *Lecidea cancriformis* (36%) and *Carbonea vorticosa* (13%) in sandstone (Figure 2F). The first two species are endemic to Antarctica.

### 3.2. Unique Species Divide the Fungal Communities Associated with Sandstone and Granite

**Figure 3:**
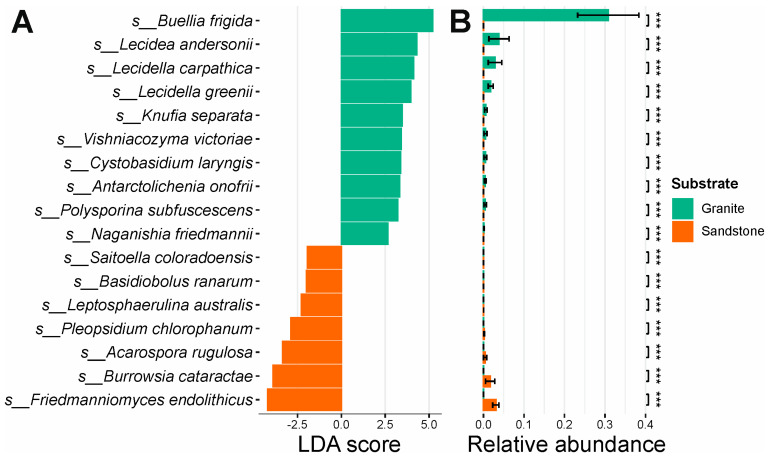
LEfSe analysis. (**A**) LEfSe between granite and sandstone relative to fungal species. (**B**) Differential abundance between the most segregative species for different rock substrates. *** = *p*-value < 0.001.

The effect size of the separation between the granite and sandstone fungal diversity analyses showed species with the highest discriminatory power between the two types of rocks substrate. Statistically significant results (LDA > 2, *p*-value < 0.001) are reported in the Supplementary Materials (Table S1). The fungal species with higher segregative values and statistically higher abundance between the two groups are as follows: *Buellia frigida*, *Lecidea andersonii*, *Lecidella carpathica*, *Lecidella grenii*, *Knufia separata*, *Vishniacozyma victoriae*, *Cystobasidium laringis*, *Antarctolichenia onofrii*, *Polysporina subfuscescens* and *Naganishia friedmanii* for granite, and *Friedmmanniomyces endolithicus*, *Burrowsia cataractae*, *Acarospora rugulosa*, *Pleopsidium chlorophanum*, *Leptosphaerulina australis*, *Basidiobolus ranarum* and *Saitoella coloradoensis* for sandstone (Figure 3A,B).

### 3.3. Granite Hosts Higher Fungal Diversity than Sandstone

**Figure 4:**
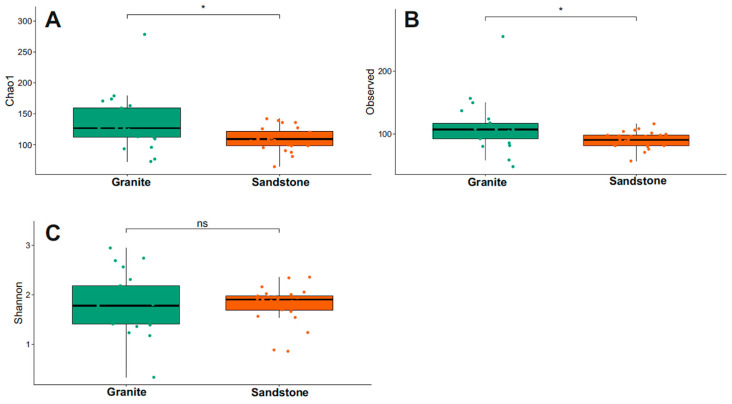
Comparison of alpha diversity using Chao1, Observed and Shannon indexes of fungi (**A**–**C**) between granite and sandstone substrates. Statistical support was tested via the Wilcoxon test, indicated as * *p* < 0.05. ns = not statistically significant.

The Chao1 index for fungi diversity is visualized in Figure 4 and Supplementary Table S1. Granite presented the highest Chao1 and Observed values between the two substrates (Wilcoxon test, *p*-value < 0.05). The Shannon index indicated that the biodiversity was the same between the two groups considered (*p*-value > 0.05).

### 3.4. Influence of Rock Type and Geographical Location on Species Diversity

**Figure 5:**
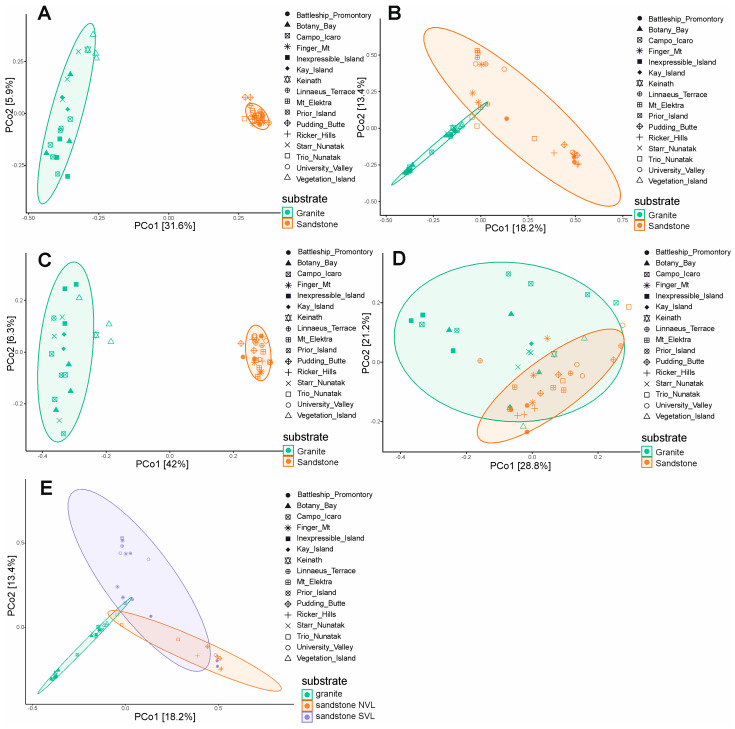
Beta diversity and principal component analysis (PCoA) based on Jaccard and Bray–Curtis indexes, and unweighted UniFrac and weighted UniFrac distance, including ellipse of 95% confidence interval. Jaccard distance (**A**), Bray–Curtis (**B**), unweighted UniFrac (**C**), weighted UniFrac (**D**), Bray–Curtis considering sandstone typology (**E**).

The Jaccard and Bray–Curtis indexes, and unweighted UniFrac and weighted UniFrac distances, were used to assess the beta diversity and the differences in fungal assemblages among the two types of substrates. The differences between granite and sandstone are significant (*p*-value < 0.001) when selecting specific species for each rock type (31% and 41% r^2^, respectively) and when using presence/absence-based metrics (Jaccard and unweighted UniFrac) (Figure 5A,C). However, when abundance is considered (Bray–Curtis and weighted UniFrac), the geographic location or the sandstone geology (40% and 41% r^2^) appear to have a greater influence on diversity than rock type (14% and 11% r^2^) (Figure 5B,D; Supplementary Table S1). Additionally, we observed a significant difference between the Beacon sandstone Supergroup from the southern sites and sedimentary rocks collected in Northern Victoria Land (*p*-value < 0.001). This finding supports the greater heterogeneity among sandstone sites when using abundance-based metrics (Bray–Curtis, weighted UniFrac) (Figure 5E).

## 4. Discussion

In this study, we retrieved raw sequence data from two publicly available datasets [17,18] of endolithic fungal communities colonizing sandstone and granite rocks in Victoria Land to implement our comparative analysis in a novel context, focusing specifically on rock typology as a shaping factor in endolithic fungal assemblages.

The fungal diversity associated with both granite and sandstone communities in the examined samples was primarily composed of Ascomycota phylum (Figure 2A). This finding aligns with previous studies on lichen-dominated endolithic communities inhabiting different rock types [17,32,33,34,35]. The most prevalent fungal class identified among the two types of substrates is Lecanoromycetes (Figure 2B), which was observed in comparable percentages within the cryptoendolithic communities of Victoria Land [17]; this included Caliciales, Lecideales, and Lecanorales Acarosporales orders for both the substrates (Figure 3B). Three fungal classes (Lecanoromycetes, Tremellomycetes, and Dothideomycetes) accounted for almost the entire relative abundance in sandstone samples. Although this is the first time they have been found in such dominance, they have already been identified in previous studies [32,34,35]. At the species taxonomic level, the data from granite samples are consistent with recent findings [17]. Regarding sandstone, nearly 50% of the total species are represented by *Lecidea cancriformis*, *Carbonea vorticosa*, and *Lecanora fuscobrunnea* (Figure 2F). The occurrence of these species in sandstone has already been reported in Antarctica; these species were among those more strongly associated with rock substrates compared to soil in the same areas [36]. One of the most striking differences in terms of community composition is the absence of *Buellia frigida* in sandstone. *Buellia frigida* is known to be one of the most abundant lichen-forming fungi found on both inland and coastal Antarctic granite [37,38]. It has also been detected within granite substrates, typically in close association with *Lecidea cancriformis* [17,39], an endolithic lichen capable of breaking down granite creating cracks (chasmoendolithism) that facilitates the penetration of *Buellia frigida*, typically an epilithic crustose endemic species in Antarctica, into the rock. In colonized sandstone, however, no fractures are observed in the rock matrix, and colonization takes place exclusively within the airspaces of porous sedimentary rocks, preventing the entrance of fungi that are not strictly capable of cryptoendolithic development, such as *Buellia frigida*. For the same reason, the high abundance of *Carbonea vorticosa* observed in sandstone may suggest the cryptoendolithic growth capacity of this species. *Buellia frigida*, *Lecidea andersonii*, *Lecidea carpathica*, *Lecidella grenii*, and *Polysporina subfuscescens* were also strongly correlated with the granite substrate, as confirmed via the LEfSe method (Figure 3). This supports previous observations suggesting that the association between these lichens and granite is likely driven both by the presence of crevices created by chasmoendolithic organisms or physical events and by the specific environmental conditions of the sampling sites, which allow their establishment and growth. *Naganishia friedmanni* has been frequently found in both endolithic microbial communities and lichens in Antarctica [40,41]. *Cystobasidium laringis* has been reported in various cold environments, including Arctic regions, as well as in soils and ice samples collected at Concordia Station in Antarctica [42,43,44]. Species of the genus *Cystobasidium* isolated from polar habitats exhibit psychrophilic or psychrotolerant traits, enabling them to thrive at the low temperatures typical of their native environments. *Vishniacozyma victoriae* is among the most frequently isolated yeast species in both polar and subpolar regions [45,46]. Although *Antarctolichenia onofrii* (Lichenostigmatales) was initially isolated from sandstone [47], it was found to be strongly associated with granite in the present study and in recent observations [17]. Despite being easily isolated, this fungus quickly loses vitality in pure culture, indicating a strict dependency on nutrients supplied by its close association with other organisms in natural endolithic communities. On the other hand, the species *Acarospora rugulosa*, *Burrowsia cataractae*, *Friedmanniomyces endolithicus* and *Pleopsidium chlorophanum* appear to be associated with the sandstone substrate. *Friedmanniomyces endolithicus* is an extremophilic and poly-extremotolerant fungus highly adapted to the Antarctic environment; it is known as one of the main cryptoendolithic colonizers. It was first described in Antarctic sandstone samples collected in Victoria Land and has emerged as the black fungus most frequently isolated from Antarctic sandstone [32,48]. *Burrowsia cataractae* was isolated from Mpumalanga, South Africa [49]. It was described as a new genus and species based on a single specimen and strain, which is not ideal for the formal establishment of a new taxon. It belongs to the family *Caliciaceae* and shows close phylogenetic affinity to the genus *Buellia*, a taxon that is well represented in Antarctic environments. It is therefore plausible that *Burrowsia cataractae* may, in fact, represent a currently undescribed species of *Buellia*. Further taxonomic and phylogenetic investigations are needed to confirm this hypothesis. *Acarospora rugulosa* and *Pleopsidium chlorophanum* have previously been reported in Antarctica. These taxa may represent lichen species with hitherto unknown cryptoendolithic capabilities, potentially explaining their association with sandstone substrates [17].

Our results reveal that fungal richness, as estimated by the Chao1 index and the number of observed ASVs, is significantly higher in granite than in sandstone samples. This suggests that granite may host a more taxonomically diverse fungal community, possibly due to its structural heterogeneity and the availability of multiple microhabitats within fissures and cracks. However, the Shannon index did not differ significantly between the two substrates, indicating a comparable level of community evenness and suggesting that, despite differences in species richness, the overall biodiversity (in terms of both abundance and distribution) remains similar. An analysis of beta diversity provides further insight into the role of substrate and environmental variables in shaping fungal assemblages. Presence/absence-based metrics (Jaccard and unweighted UniFrac) revealed significant compositional differences between granite and sandstone, supporting the hypothesis that substrate type acts as a strong environmental filter influencing the presence of distinct fungal taxa. This is consistent with previous studies showing that the physical and chemical properties of rocks strongly affect microbial colonization and survival strategies in Antarctic lithic environments [16,17]. Interestingly, abundance-based metrics (Bray–Curtis and weighted UniFrac) highlighted the clear separation of sandstone samples collected from locations in Northern Victoria Land (NVL: Trio Nunatak, Ricker Hills and Pudding Butte) from those from Southern Victoria Land (SVL), with the only exception being two specimens from Battleship Promontory. The sandstones of the Beacon Supergroup in the McMurdo Dry Valleys, from which cryptoendolithic lichen communities were first discovered and described [13], are well known for their extremely homogeneous texture and scarce matrices. Instead, the outcrops characterizing Northern Victoria Land are richer in matrices between the orthoquartzite grains [50]. However, it is not possible at present to establish with certainty which parameter—between geography or sandstone typology—exerts a greater influence on community composition. This pattern reflects the complex interplay between deterministic and stochastic processes in shaping microbial communities under extreme environmental constraints. The greater fungal species richness observed in granite compared to sandstone is consistent with previous findings on bacterial communities [16]. Recent studies (e.g., [17]) and our current findings suggest that granite can support well-structured and diverse fungal assemblages, potentially facilitated by chasmoendolithic colonization, where lichen-forming and free-living fungi exploit cracks and fractures to establish symbiotic associations. Overall, these findings emphasize the importance of combining both presence/absence- and abundance-based analyses to understand microbial diversity patterns in Antarctic rock environments. They also underscore the necessity of considering microhabitat characteristics and geographic variability in future studies investigating the ecological drivers of lithic microbial communities.

## 5. Conclusions

This study provides new insights emerging from comparing the composition and diversity of fungal communities inhabiting Antarctic granite and sandstone substrates. Our findings confirm that, similarly to reports regarding bacteria, rock type plays a crucial role in shaping fungal assemblages, with granite supporting higher species richness and a distinct set of taxa compared to sandstone. Indicator fungal species related to the two rock typologies considered, sandstone (mainly the endemic black fungus Friedmanniomyces endolithicus) and granite (mainly the endemic lichen *Buellia frigida*), have been highlighted. While presence/absence metrics highlight substrate-specific community structures, abundance-based analyses reveal the influence of local environmental conditions, that cannot, at present, be addressed with certainty, on the different nature of sandstones in NVL and SVL sites or other environmental parameters. These results underscore the complexity of microbial colonization in extreme environments and the importance of integrating multiple diversity metrics and ecological variables when assessing fungal biodiversity in Antarctic lithic ecosystems. Further analyses implementing the samples considered would be necessary to reveal whether the presence of specific taxa and their relative abundance are mainly determined by rock type (specifically for sandstone) or modulated by local environmental factors such as microclimate, altitude, geographic distances or rock weathering stage.

## Figures and Tables

**Table 1:** Metadata related to the dataset analyzed and the number of samples considered from each type of rock substrate. NVL = Northern Victoria Land; SVL = Southern Victoria Land.

Locality	Coordinates	Endolithic Communities (Granite)	Endolithic Communities (Sandstone)
Botany Bay	−77.005433, 162.534183	3	0
Inexpressible Island	−74.89265, 163.7377	3	0
Vegetation Island	−74.7874, 163.641933	3	0
Prior Island	−75.691667, 162.878611	3	0
Kay Island	−74.070583, 165.316533	2	0
Starr Nunatak	−75.898767, 162.594683	3	0
Mt Keinath	−74.550117, 164.053567	1	0
Campo Icaro	−74.709367, 164.095967	3	0
Battleship Promontory	−76.9011, 160.9102	0	3
Finger Mt	−77.7503, 160.7457	0	3
Linnaeus Terrace	−77.5978, 161.0067	0	3
Mt Elektra	−77.4911, 160.9046	0	3
Pudding Butte	−75.8584, 159.9738	0	3
Ricker Hills	−75.7041, 159.2276	0	3
Trio Nunatak	−75.4824, 159.5912	0	3
University Valley	−77.8726, 160.7553	0	3

## Data Availability

All data generated and used in this study are available on NCBI under the BioProject ID PRJNA1095873 for granite and PRJNA453198 for sandstone.

## Supplementary Materials

The following supporting information can be downloaded at https://www.mdpi.com/article/10.3390/life15071028/s1. Figure S1: Species accumulation curves considering sampling sites; Table S1: Statistics.
