# Supplementary figures and images for: Abundance and Diversity of Endolithic Fungal Assemblages in Granite and Sandstone from Victoria Land, Antarctica
Source: Life (Basel). 2025 Jun 27;15(7):1028. doi: 10.3390/life15071028 (PMC12299835; doi:10.3390/life15071028)

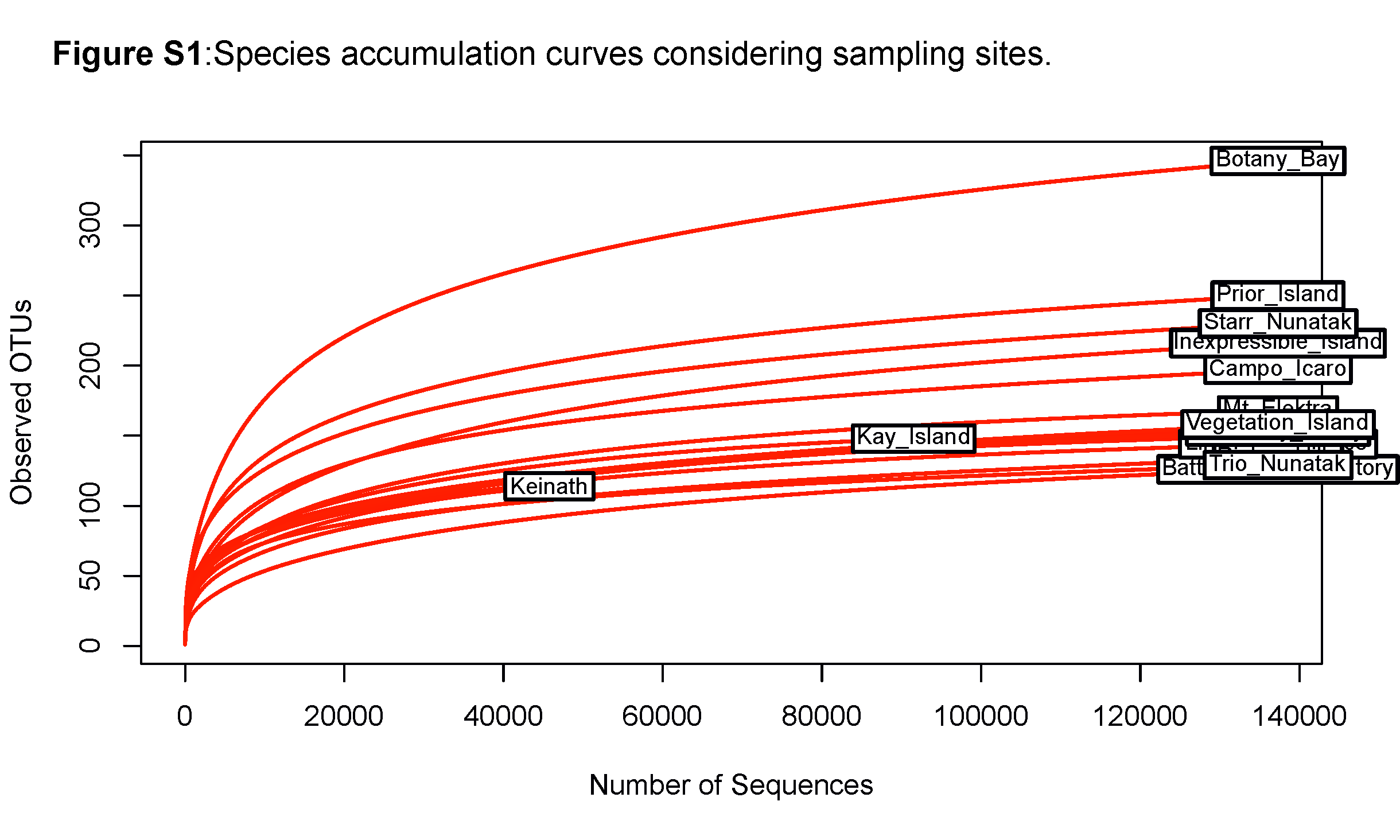

Supplement: Supplementary file 1 [file life-15-01028-s001.zip › Figure S1.tiff]
